# (2,3,5,6-Tetra­fluoro­phenolato-κ*O*)(5,10,15,20-tetra­phenyl­porphyrinato)iron(III)

**DOI:** 10.1107/S160053681302607X

**Published:** 2013-09-28

**Authors:** Nan Xu, Douglas R. Powell, George B. Richter-Addo

**Affiliations:** a Department of Chemistry and Biochemistry, University of Oklahoma, 101 Stephenson Pkwy, Norman, OK 73019, USA

## Abstract

The title compound, [Fe(C_44_H_28_N_4_)(C_6_HF_4_O)], is a porphyrin complex with iron(III) in fivefold coordination with a tetra­fluoro­phenolate group as the axial ligand. The Fe atom and the phenolate ligand are disordered across the porphyrin ring with the two phenolates appearing to be roughly related by a center of symmetry. The occupancies of the two phenolate groups refined to 0.788 (3) for the major component and 0.212 (3) for the minor component. The structure shows extraordinary Fe displacements of 0.488 (4) (major) and 0.673 (4) Å (minor) from the 24-atom mean plane of the porphyrin. The Fe—N_p_ distances range from 2.063 (4) to 2.187 (6) Å and the Fe—O distances are 1.903 (5) Å for major component and 1.87 (2) Å for minor component. The four phenyl groups attached to the porphyrin ring form dihedral angles of 63.4 (4), 49.6 (4), 62.4 (4), and 63.3 (4)° (in increasing numerical order) with the three nearest C atoms of the porphyrin ring. The major and minor component phenolate groups form dihedral angles of 24.9 (4)° and 24.8 (4)°, respectively, with the four porphyrin N atoms. The Fe⋯Fe distance between the two iron(III) atoms of adjacent porphyrin mol­ecules is 6.677 (3) Å. No close inter­molecular inter­action was observed. The crystal studied was twinned by inversion, with a major–minor component ratio of 0.53 (3):0.47 (3).

## Related literature

For the function and structure of catalase, see: Nicholls *et al.* (2001 ▶). For the structures of other related ferric phenolate porphyrin derivatives, see: Xu *et al.* (2013 ▶); Chaudhary *et al.* (2010 ▶); Ueyama *et al.* (1998 ▶); Kanamori *et al.* (2005 ▶); Byrn *et al.* (1993 ▶). For the preparation of the [(TPP)Fe]_2_O (TPP is tetraphenylporphyrin) complex, see: Helms *et al.* (1986 ▶).
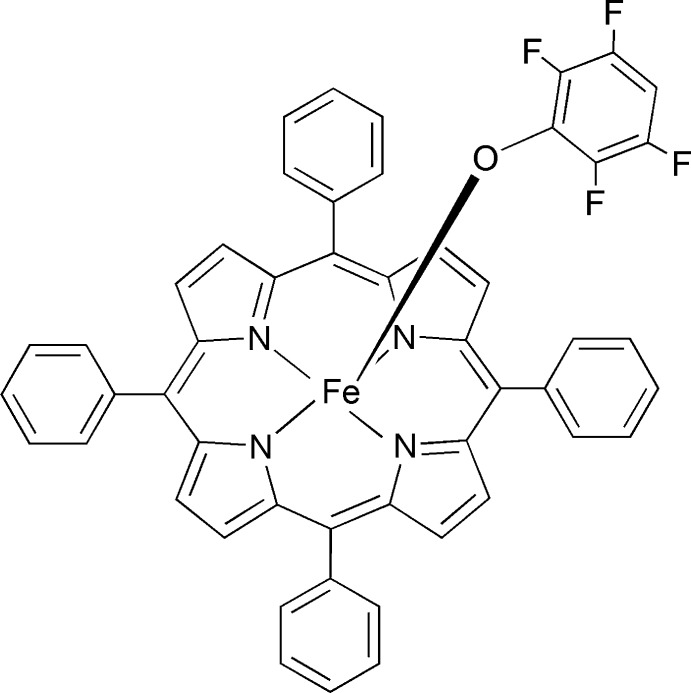


## Experimental

### 

#### Crystal data

[Fe(C_44_H_28_N_4_)(C_6_HF_4_O)]
*M* *_r_* = 833.62
Monoclinic, 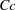

*a* = 22.287 (4) Å
*b* = 12.676 (2) Å
*c* = 13.339 (2) Å
β = 98.510 (4)°
*V* = 3727 (2) Å^3^
*Z* = 4
Mo *K*α radiation
μ = 0.47 mm^−1^
*T* = 100 K
0.34 × 0.16 × 0.14 mm

#### Data collection

Bruker APEX CCD diffractometer
Absorption correction: multi-scan (*SADABS*; Bruker, 2001 ▶) *T*_min_ = 0.856, *T*_max_ = 0.937
34669 measured reflections
9209 independent reflections
6448 reflections with *I* > 2σ(*I*)
*R*_int_ = 0.087

#### Refinement

*R*[*F* ^2^ > 2σ(*F* ^2^)] = 0.056
*wR*(*F* ^2^) = 0.116
*S* = 1.01
9209 reflections
651 parameters
240 restraints
H-atom parameters constrained
Δρ_max_ = 0.28 e Å^−3^
Δρ_min_ = −0.40 e Å^−3^

### 

Data collection: *SMART* (Bruker, 2007 ▶); cell refinement: *SAINT* (Bruker, 2007 ▶); data reduction: *SAINT*; program(s) used to solve structure: *SHELXS97* (Sheldrick, 2008 ▶); program(s) used to refine structure: *SHELXL2013* (Sheldrick, 2008 ▶); molecular graphics: *SHELXTL* (Sheldrick, 2008 ▶); software used to prepare material for publication: *SHELXL2013*.

## Supplementary Material

Crystal structure: contains datablock(s) I, general. DOI: 10.1107/S160053681302607X/pk2492sup1.cif

Structure factors: contains datablock(s) I. DOI: 10.1107/S160053681302607X/pk2492Isup2.hkl

Additional supplementary materials:  crystallographic information; 3D view; checkCIF report

## Figures and Tables

**Fig. 1.**
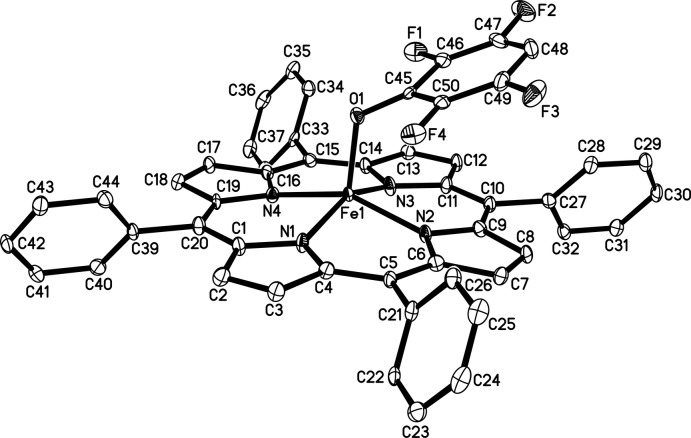
The molecular structure of (TPP)Fe(OC6HF4) with displacement ellipsoids drawn at the 35% probability level. H atoms and the minor disorder component are omitted for clarity.

## supplementary crystallographic information

### 1. Comment

Metalloporphyrin complexes with phenolate ligands are potential structural models for heme catalase (Chaudhary *et al.*, 2010; Nicholls *et al.*, 2001). Strouse and coworkers have reported the crystal structures of several iron phenoxide porphyrin complexes, showing their ability to accommodate various small molecules in the clathrate lattice (Byrn *et al.*, 1993). In this paper, we report the structure of (5,10,15,20-tetraphenylporphyrinato)(2,3,5,6-tetrafluorophenolato)iron(III).

The Fe and the phenolate ligand were disordered across the porphyrin plane. The occupancy of the Fe and axial phenolate ligand refined to 0.788 (3) and 0.212 (3) for the primed and unprimed atoms, respectively. The molecular structure of (5,10,15,20-tetraphenylporphyrinato)(2,3,5,6-tetrafluorophenolato)iron(III) is shown in Fig. 1. The Fe atom is displaced by 0.488 (4) Å (major) and 0.673 (4) Å (minor) from the 24-atom mean porphyrin plane toward the tetrafluorophenolate anion. The average Fe—N_p_ distances are 2.075 (4) Å (major) and 2.127 (6) Å (minor). These long Fe—N_p_ bonds are consistent with the large displacement of the iron centers. The Fe—O—C bond angles are 122.4 (7)° and 123 (3)° for the major and minor components, respectively. The Fe—O distances of both disordered components (1.903 (5) Å for major and 1.87 (2) Å for minor) are similar to the Fe—O bond distances in other iron phenolate porphyrin complexes reported previously (Xu *et al.*, 2013; Chaudhary *et al.*, 2010; Ueyama *et al.*, 1998, Kanamori *et al.*, 2005; Byrn *et al.*, 1993). The structure is twinned by inversion, with a major:minor component ratio of 0.53 (3):0.47 (3).

### 2. Experimental

To a CH_2_Cl_2_ solution (20 ml) of [(TPP)Fe]_2_O (Helms *et al.*, 1986) (0.025 g, 0.018 mmol) was added 2,3,5,6-tetrafluorophenol (0.045 g, 0.271 mmol) (purchased from Aldrich Chemical Company and used as received) under N_2_. After stirring for 1 h, the color of the solution changed from green brown to red. The solution was reduced to 2 ml and 10 ml hexane was added. The resulting dark brown precipitate was collected by filtration and dried under vacuum. A suitable rod-shaped crystal was grown by slow evaporation of a CH_2_Cl_2_-hexane (1:2) solution of the complex at room temperature under N_2_.

### 3. Refinement

The iron and the phenolate ligand were disordered across the porphyrin ring. The occupancies of the metal and axial ligand refined to 0.787 (3) and 0.213 (3) for the primed and unprimed atoms. Rigid-body restraints were applied to the displacement parameters of both phenolate disorder components. The hydrogens were located by geometry assuming C—H distances of 0.95 Å, and were refined with a riding model. The hydrogen displacement parameters were set to 1.2 times the isotropic equivalent of the bonded carbon. The structure was twinned by inversion, with a major:minor component ratio of 0.53 (3):0.47 (3).

### Crystal data

**Table:**

[Fe(C_44_H_28_N_4_)(C_6_HF_4_O)]	*F*(000) = 1708
*M**_r_* = 833.62	*D*_x_ = 1.486 Mg m^−^^3^
Monoclinic, *C**c*	Mo *K*α radiation, λ = 0.71073 Å
*a* = 22.287 (4) Å	Cell parameters from 7386 reflections
*b* = 12.676 (2) Å	θ = 2.3–27.1°
*c* = 13.339 (2) Å	µ = 0.47 mm^−^^1^
β = 98.510 (4)°	*T* = 100 K
*V* = 3727 (2) Å^3^	Rod, black
*Z* = 4	0.34 × 0.16 × 0.14 mm

### Data collection

**Table:**

Bruker APEX CCD diffractometer	6448 reflections with *I* > 2σ(*I*)
φ and ω scans	*R*_int_ = 0.087
Absorption correction: multi-scan (*SADABS*; Bruker, 2001)	θ_max_ = 28.4°, θ_min_ = 1.9°
*T*_min_ = 0.856, *T*_max_ = 0.937	*h* = −29→29
34669 measured reflections	*k* = −16→16
9209 independent reflections	*l* = −17→17

### Refinement

**Table:**

Refinement on *F*^2^	Secondary atom site location: difference Fourier map
Least-squares matrix: full	Hydrogen site location: inferred from neighbouring sites
*R*[*F*^2^ > 2σ(*F*^2^)] = 0.056	H-atom parameters constrained
*wR*(*F*^2^) = 0.116	*w* = 1/[σ^2^(*F*_o_^2^) + (0.046*P*)^2^] where *P* = (*F*_o_^2^ + 2*F*_c_^2^)/3
*S* = 1.01	(Δ/σ)_max_ = 0.002
9209 reflections	Δρ_max_ = 0.28 e Å^−^^3^
651 parameters	Δρ_min_ = −0.40 e Å^−^^3^
240 restraints	Absolute structure: Refined as an inversion twin.
Primary atom site location: structure-invariant direct methods	Absolute structure parameter: 0.47 (3)

### Special details

**Table:**

Refinement. Refined as a 2-component inversion twin.

### Fractional atomic coordinates and isotropic or equivalent isotropic displacement parameters (Å^2^)

**Table:**

	*x*	*y*	*z*	*U*_iso_*/*U*_eq_	Occ. (<1)
Fe1	0.51168 (5)	0.48729 (6)	0.54296 (7)	0.0126 (3)	0.788 (3)
F1	0.5831 (2)	0.2280 (4)	0.6516 (3)	0.0324 (10)	0.788 (3)
F2	0.5280 (3)	0.0706 (3)	0.7383 (4)	0.0485 (13)	0.788 (3)
F3	0.4033 (2)	0.3187 (5)	0.8728 (3)	0.0455 (13)	0.788 (3)
F4	0.4609 (2)	0.4764 (4)	0.7911 (3)	0.0293 (10)	0.788 (3)
O1	0.5484 (2)	0.4354 (4)	0.6714 (4)	0.0183 (11)	0.788 (3)
Fe1'	0.48973 (16)	0.5111 (3)	0.4615 (3)	0.0192 (13)	0.212 (3)
F1'	0.5413 (8)	0.5229 (15)	0.2136 (12)	0.032 (4)	0.212 (3)
F2'	0.5950 (9)	0.6856 (18)	0.1327 (13)	0.051 (5)	0.212 (3)
F3'	0.4701 (11)	0.9297 (14)	0.2697 (14)	0.054 (5)	0.212 (3)
F4'	0.4163 (9)	0.7719 (15)	0.3514 (15)	0.048 (5)	0.212 (3)
O1'	0.4521 (10)	0.5641 (16)	0.3375 (16)	0.029 (4)	0.212 (3)
N1	0.4870 (2)	0.6413 (3)	0.5683 (3)	0.0191 (11)	
N2	0.4222 (2)	0.4470 (3)	0.5455 (3)	0.0172 (10)	
N3	0.5164 (2)	0.3543 (3)	0.4520 (4)	0.0211 (11)	
N4	0.58083 (19)	0.5504 (3)	0.4727 (3)	0.0175 (10)	
C1	0.5238 (3)	0.7312 (4)	0.5686 (4)	0.0188 (13)	
C2	0.4945 (3)	0.8192 (4)	0.6093 (4)	0.0218 (13)	
H2	0.5097	0.8892	0.6171	0.026*	
C3	0.4410 (3)	0.7838 (4)	0.6347 (4)	0.0206 (13)	
H3	0.4123	0.8240	0.6647	0.025*	
C4	0.4361 (3)	0.6737 (4)	0.6076 (4)	0.0177 (13)	
C5	0.3861 (2)	0.6108 (4)	0.6183 (4)	0.0174 (12)	
C6	0.3793 (2)	0.5052 (4)	0.5863 (4)	0.0179 (11)	
C7	0.3263 (3)	0.4425 (4)	0.5904 (4)	0.0185 (12)	
H7	0.2904	0.4643	0.6153	0.022*	
C8	0.3366 (3)	0.3463 (4)	0.5522 (4)	0.0177 (12)	
H8	0.3095	0.2881	0.5452	0.021*	
C9	0.3963 (2)	0.3488 (4)	0.5244 (4)	0.0169 (12)	
C10	0.4228 (3)	0.2629 (4)	0.4820 (4)	0.0177 (12)	
C11	0.4796 (3)	0.2674 (4)	0.4479 (4)	0.0185 (12)	
C12	0.5068 (2)	0.1798 (4)	0.4036 (4)	0.0204 (13)	
H12	0.4907	0.1105	0.3944	0.024*	
C13	0.5594 (3)	0.2147 (4)	0.3775 (4)	0.0199 (13)	
H13	0.5870	0.1746	0.3449	0.024*	
C14	0.5665 (3)	0.3240 (4)	0.4078 (4)	0.0179 (12)	
C15	0.6154 (3)	0.3882 (4)	0.3936 (4)	0.0166 (12)	
C16	0.6219 (2)	0.4925 (4)	0.4257 (4)	0.0166 (11)	
C17	0.6735 (3)	0.5583 (4)	0.4158 (4)	0.0186 (12)	
H17	0.7082	0.5378	0.3870	0.022*	
C18	0.6638 (3)	0.6535 (4)	0.4544 (4)	0.0187 (13)	
H18	0.6903	0.7125	0.4577	0.022*	
C19	0.6060 (3)	0.6501 (4)	0.4899 (4)	0.0162 (12)	
C20	0.5796 (3)	0.7352 (4)	0.5334 (4)	0.0178 (12)	
C21	0.3355 (2)	0.6582 (4)	0.6651 (4)	0.0182 (12)	
C22	0.3016 (2)	0.7416 (4)	0.6170 (4)	0.0177 (12)	
H22	0.3123	0.7699	0.5561	0.021*	
C23	0.2526 (3)	0.7838 (4)	0.6569 (4)	0.0229 (14)	
H23	0.2301	0.8407	0.6237	0.027*	
C24	0.2368 (3)	0.7417 (5)	0.7463 (4)	0.0257 (14)	
H24	0.2031	0.7694	0.7737	0.031*	
C25	0.2701 (3)	0.6601 (5)	0.7946 (4)	0.0267 (14)	
H25	0.2592	0.6320	0.8555	0.032*	
C26	0.3194 (3)	0.6182 (4)	0.7555 (4)	0.0207 (13)	
H26	0.3423	0.5624	0.7900	0.025*	
C27	0.3888 (3)	0.1617 (4)	0.4690 (4)	0.0187 (12)	
C28	0.3766 (3)	0.1034 (4)	0.5531 (4)	0.0220 (13)	
H28	0.3896	0.1289	0.6198	0.026*	
C29	0.3454 (3)	0.0077 (4)	0.5388 (4)	0.0227 (14)	
H29	0.3368	−0.0309	0.5959	0.027*	
C30	0.3271 (3)	−0.0312 (4)	0.4428 (4)	0.0218 (13)	
H30	0.3076	−0.0978	0.4338	0.026*	
C31	0.3374 (3)	0.0275 (4)	0.3597 (4)	0.0221 (13)	
H31	0.3234	0.0023	0.2932	0.027*	
C32	0.3679 (2)	0.1225 (4)	0.3726 (4)	0.0194 (13)	
H32	0.3748	0.1618	0.3147	0.023*	
C33	0.6656 (2)	0.3419 (4)	0.3438 (4)	0.0159 (12)	
C34	0.7000 (2)	0.2582 (4)	0.3883 (4)	0.0196 (12)	
H34	0.6903	0.2281	0.4492	0.024*	
C35	0.7478 (3)	0.2180 (4)	0.3457 (4)	0.0229 (14)	
H35	0.7707	0.1605	0.3770	0.027*	
C36	0.7626 (3)	0.2614 (5)	0.2573 (4)	0.0258 (15)	
H36	0.7962	0.2353	0.2286	0.031*	
C37	0.7277 (3)	0.3434 (5)	0.2114 (4)	0.0241 (14)	
H37	0.7373	0.3727	0.1502	0.029*	
C38	0.6791 (3)	0.3833 (4)	0.2529 (4)	0.0206 (13)	
H38	0.6551	0.4386	0.2197	0.025*	
C39	0.6127 (2)	0.8381 (4)	0.5434 (4)	0.0159 (12)	
C40	0.6245 (3)	0.8940 (4)	0.4597 (4)	0.0207 (13)	
H40	0.6121	0.8658	0.3939	0.025*	
C41	0.6540 (2)	0.9903 (4)	0.4696 (4)	0.0225 (13)	
H41	0.6620	1.0275	0.4112	0.027*	
C42	0.6720 (3)	1.0323 (4)	0.5659 (5)	0.0229 (14)	
H42	0.6913	1.0993	0.5731	0.028*	
C43	0.6618 (3)	0.9773 (4)	0.6502 (4)	0.0223 (12)	
H43	0.6746	1.0055	0.7159	0.027*	
C44	0.6325 (3)	0.8792 (4)	0.6393 (4)	0.0204 (13)	
H44	0.6261	0.8405	0.6978	0.024*	
C45	0.5240 (6)	0.3592 (9)	0.7168 (9)	0.0138 (17)	0.788 (3)
C46	0.5390 (4)	0.2499 (7)	0.7079 (7)	0.0218 (16)	0.788 (3)
C47	0.5099 (5)	0.1722 (8)	0.7515 (8)	0.0289 (19)	0.788 (3)
C48	0.4650 (4)	0.1910 (8)	0.8083 (7)	0.0336 (15)	0.788 (3)
H48	0.4456	0.1353	0.8386	0.040*	0.788 (3)
C49	0.4491 (4)	0.2959 (8)	0.8195 (7)	0.0281 (18)	0.788 (3)
C50	0.4784 (6)	0.3757 (8)	0.7765 (9)	0.0175 (18)	0.788 (3)
C45'	0.473 (3)	0.644 (4)	0.291 (4)	0.030 (6)	0.212 (3)
C46'	0.517 (3)	0.630 (4)	0.234 (4)	0.032 (9)	0.212 (3)
C47'	0.5473 (18)	0.709 (3)	0.191 (3)	0.033 (6)	0.212 (3)
C48'	0.5329 (19)	0.811 (4)	0.200 (3)	0.046 (5)	0.212 (3)
H48'	0.5526	0.8657	0.1683	0.055*	0.212 (3)
C49'	0.487 (2)	0.833 (4)	0.259 (4)	0.039 (9)	0.212 (3)
C50'	0.4568 (19)	0.747 (3)	0.309 (3)	0.031 (7)	0.212 (3)

### Atomic displacement parameters (Å^2^)

**Table:**

	*U*^11^	*U*^22^	*U*^33^	*U*^12^	*U*^13^	*U*^23^
Fe1	0.0129 (5)	0.0094 (4)	0.0163 (5)	−0.0022 (5)	0.0048 (3)	0.0001 (5)
F1	0.034 (3)	0.031 (3)	0.032 (3)	0.010 (2)	0.0049 (19)	−0.0036 (19)
F2	0.071 (4)	0.017 (2)	0.051 (3)	−0.003 (2)	−0.013 (3)	0.0044 (19)
F3	0.026 (3)	0.089 (4)	0.023 (2)	−0.015 (2)	0.0084 (18)	0.004 (2)
F4	0.028 (3)	0.035 (2)	0.026 (2)	0.009 (2)	0.0065 (19)	−0.0053 (19)
O1	0.021 (3)	0.017 (2)	0.018 (2)	−0.005 (2)	0.0043 (19)	0.0018 (19)
Fe1'	0.014 (2)	0.025 (2)	0.020 (2)	0.0005 (19)	0.0077 (16)	−0.0024 (17)
F1'	0.036 (10)	0.038 (6)	0.020 (10)	0.009 (7)	−0.005 (7)	−0.011 (6)
F2'	0.046 (10)	0.078 (12)	0.030 (9)	0.003 (8)	0.008 (7)	0.004 (9)
F3'	0.076 (13)	0.028 (6)	0.055 (12)	0.008 (7)	−0.005 (9)	0.003 (6)
F4'	0.049 (10)	0.042 (10)	0.055 (11)	0.011 (7)	0.012 (8)	−0.009 (8)
O1'	0.035 (10)	0.026 (7)	0.026 (7)	0.008 (7)	0.000 (5)	−0.001 (6)
N1	0.014 (3)	0.015 (2)	0.030 (3)	−0.0036 (19)	0.010 (2)	−0.0033 (19)
N2	0.017 (2)	0.010 (2)	0.027 (3)	−0.0022 (18)	0.008 (2)	−0.0024 (18)
N3	0.020 (3)	0.011 (2)	0.035 (3)	−0.0016 (19)	0.013 (2)	−0.0017 (19)
N4	0.013 (2)	0.014 (2)	0.026 (3)	−0.0004 (18)	0.005 (2)	−0.0007 (18)
C1	0.018 (3)	0.013 (3)	0.026 (3)	−0.002 (2)	0.008 (3)	−0.001 (2)
C2	0.017 (3)	0.020 (3)	0.028 (3)	0.000 (3)	0.004 (3)	−0.005 (2)
C3	0.019 (3)	0.016 (3)	0.028 (3)	0.001 (2)	0.007 (3)	−0.006 (2)
C4	0.016 (3)	0.014 (3)	0.024 (3)	0.001 (2)	0.005 (2)	−0.004 (2)
C5	0.012 (3)	0.017 (3)	0.024 (3)	−0.001 (2)	0.006 (2)	0.003 (2)
C6	0.017 (3)	0.016 (3)	0.022 (3)	0.003 (2)	0.007 (2)	−0.001 (2)
C7	0.014 (3)	0.022 (3)	0.021 (3)	−0.001 (2)	0.007 (2)	−0.001 (2)
C8	0.017 (3)	0.017 (3)	0.021 (3)	−0.004 (2)	0.009 (2)	0.002 (2)
C9	0.018 (3)	0.015 (3)	0.020 (3)	0.001 (2)	0.008 (2)	0.001 (2)
C10	0.018 (3)	0.012 (3)	0.024 (3)	−0.004 (2)	0.003 (2)	−0.001 (2)
C11	0.014 (3)	0.010 (3)	0.032 (3)	−0.002 (2)	0.006 (3)	−0.003 (2)
C12	0.021 (3)	0.011 (3)	0.030 (3)	−0.005 (2)	0.007 (3)	−0.002 (2)
C13	0.019 (3)	0.016 (3)	0.025 (3)	0.002 (2)	0.005 (3)	−0.001 (2)
C14	0.016 (3)	0.015 (3)	0.024 (3)	−0.003 (2)	0.008 (2)	0.001 (2)
C15	0.017 (3)	0.014 (3)	0.019 (3)	0.001 (2)	0.004 (2)	0.001 (2)
C16	0.014 (3)	0.019 (3)	0.019 (3)	−0.003 (2)	0.008 (2)	0.004 (2)
C17	0.016 (3)	0.019 (3)	0.023 (3)	−0.004 (2)	0.010 (2)	0.003 (2)
C18	0.016 (3)	0.019 (3)	0.021 (3)	−0.004 (2)	0.003 (2)	−0.003 (2)
C19	0.014 (3)	0.012 (3)	0.023 (3)	−0.006 (2)	0.003 (2)	0.002 (2)
C20	0.018 (3)	0.014 (3)	0.023 (3)	−0.003 (2)	0.008 (2)	−0.004 (2)
C21	0.016 (3)	0.016 (3)	0.024 (3)	−0.006 (2)	0.006 (2)	−0.005 (2)
C22	0.016 (3)	0.015 (3)	0.022 (3)	−0.007 (2)	0.003 (2)	−0.001 (2)
C23	0.023 (3)	0.019 (3)	0.027 (3)	−0.001 (3)	0.000 (3)	−0.004 (2)
C24	0.021 (3)	0.029 (3)	0.028 (3)	−0.006 (3)	0.009 (3)	−0.017 (3)
C25	0.028 (4)	0.033 (4)	0.021 (3)	−0.007 (3)	0.008 (3)	−0.010 (3)
C26	0.022 (4)	0.020 (3)	0.021 (3)	−0.005 (2)	0.004 (3)	−0.004 (2)
C27	0.016 (3)	0.013 (3)	0.028 (3)	0.003 (2)	0.006 (3)	0.003 (2)
C28	0.019 (3)	0.022 (3)	0.025 (3)	−0.005 (2)	0.003 (3)	−0.001 (2)
C29	0.021 (3)	0.016 (3)	0.032 (4)	−0.004 (3)	0.008 (3)	0.000 (3)
C30	0.020 (3)	0.014 (3)	0.032 (3)	−0.007 (2)	0.005 (3)	−0.002 (2)
C31	0.021 (3)	0.021 (3)	0.024 (3)	−0.004 (3)	0.003 (2)	−0.003 (2)
C32	0.018 (3)	0.019 (3)	0.023 (3)	−0.001 (2)	0.009 (2)	0.004 (2)
C33	0.007 (3)	0.016 (3)	0.024 (3)	−0.003 (2)	0.004 (2)	−0.006 (2)
C34	0.021 (3)	0.016 (3)	0.023 (3)	−0.001 (2)	0.005 (2)	−0.003 (2)
C35	0.016 (3)	0.018 (3)	0.034 (3)	−0.001 (2)	0.004 (3)	−0.013 (3)
C36	0.014 (3)	0.034 (4)	0.031 (4)	−0.004 (3)	0.009 (3)	−0.017 (3)
C37	0.026 (4)	0.031 (3)	0.017 (3)	−0.010 (3)	0.008 (3)	−0.005 (2)
C38	0.020 (3)	0.020 (3)	0.023 (3)	−0.005 (2)	0.005 (3)	−0.001 (2)
C39	0.012 (3)	0.009 (3)	0.028 (3)	0.000 (2)	0.008 (2)	−0.001 (2)
C40	0.020 (3)	0.019 (3)	0.023 (3)	0.001 (2)	0.003 (2)	−0.001 (2)
C41	0.025 (3)	0.017 (3)	0.025 (3)	−0.003 (3)	0.004 (2)	0.006 (3)
C42	0.023 (3)	0.011 (3)	0.035 (4)	−0.004 (3)	0.003 (3)	0.001 (2)
C43	0.019 (3)	0.018 (3)	0.030 (3)	−0.002 (3)	0.005 (3)	−0.004 (2)
C44	0.022 (3)	0.016 (3)	0.024 (3)	0.000 (2)	0.004 (3)	0.000 (2)
C45	0.011 (4)	0.017 (3)	0.012 (4)	−0.001 (2)	0.001 (3)	0.001 (2)
C46	0.023 (4)	0.014 (2)	0.026 (4)	0.001 (3)	−0.002 (3)	0.001 (3)
C47	0.041 (6)	0.019 (2)	0.023 (5)	−0.006 (3)	−0.008 (4)	0.006 (3)
C48	0.039 (4)	0.035 (3)	0.024 (4)	−0.020 (3)	−0.006 (3)	0.008 (3)
C49	0.022 (4)	0.042 (3)	0.019 (4)	−0.014 (3)	0.002 (3)	0.004 (3)
C50	0.014 (4)	0.025 (3)	0.013 (4)	−0.002 (3)	0.002 (3)	−0.001 (3)
C45'	0.039 (13)	0.026 (6)	0.024 (11)	0.006 (6)	0.003 (10)	−0.001 (6)
C46'	0.037 (15)	0.033 (6)	0.024 (17)	0.002 (6)	0.001 (14)	−0.009 (6)
C47'	0.033 (11)	0.044 (6)	0.018 (12)	−0.004 (6)	−0.008 (9)	−0.003 (6)
C48'	0.048 (7)	0.042 (6)	0.047 (8)	−0.006 (5)	0.001 (5)	0.001 (5)
C49'	0.045 (14)	0.028 (6)	0.040 (19)	0.002 (7)	−0.006 (15)	0.002 (7)
C50'	0.035 (12)	0.026 (6)	0.029 (14)	0.007 (6)	−0.005 (11)	−0.005 (7)

### Geometric parameters (Å, º)

**Table:**

Fe1—O1	1.903 (5)	C20—C39	1.495 (7)
Fe1—N2	2.063 (4)	C21—C22	1.398 (7)
Fe1—N1	2.070 (4)	C21—C26	1.403 (7)
Fe1—N4	2.078 (4)	C22—C23	1.391 (8)
Fe1—N3	2.089 (4)	C22—H22	0.9500
F1—C46	1.351 (10)	C23—C24	1.399 (8)
F2—C47	1.369 (11)	C23—H23	0.9500
F3—C49	1.359 (10)	C24—C25	1.377 (8)
F4—C50	1.356 (12)	C24—H24	0.9500
O1—C45	1.302 (12)	C25—C26	1.391 (7)
Fe1'—O1'	1.87 (2)	C25—H25	0.9500
Fe1'—N4	2.074 (6)	C26—H26	0.9500
Fe1'—N3	2.083 (6)	C27—C32	1.392 (7)
Fe1'—N2	2.163 (6)	C27—C28	1.404 (7)
Fe1'—N1	2.187 (6)	C28—C29	1.396 (8)
F1'—C46'	1.50 (6)	C28—H28	0.9500
F2'—C47'	1.44 (4)	C29—C30	1.376 (7)
F3'—C49'	1.30 (5)	C29—H29	0.9500
F4'—C50'	1.18 (4)	C30—C31	1.382 (8)
O1'—C45'	1.31 (5)	C30—H30	0.9500
N1—C4	1.381 (7)	C31—C32	1.382 (7)
N1—C1	1.404 (6)	C31—H31	0.9500
N2—C9	1.384 (6)	C32—H32	0.9500
N2—C6	1.383 (6)	C33—C34	1.390 (7)
N3—C11	1.370 (6)	C33—C38	1.394 (7)
N3—C14	1.392 (7)	C34—C35	1.378 (8)
N4—C19	1.388 (6)	C34—H34	0.9500
N4—C16	1.393 (6)	C35—C36	1.384 (8)
C1—C20	1.393 (8)	C35—H35	0.9500
C1—C2	1.439 (7)	C36—C37	1.386 (8)
C2—C3	1.363 (8)	C36—H36	0.9500
C2—H2	0.9500	C37—C38	1.384 (7)
C3—C4	1.441 (7)	C37—H37	0.9500
C3—H3	0.9500	C38—H38	0.9500
C4—C5	1.394 (7)	C39—C40	1.379 (7)
C5—C6	1.407 (7)	C39—C44	1.392 (7)
C5—C21	1.493 (7)	C40—C41	1.384 (7)
C6—C7	1.432 (7)	C40—H40	0.9500
C7—C8	1.355 (7)	C41—C42	1.393 (7)
C7—H7	0.9500	C41—H41	0.9500
C8—C9	1.432 (7)	C42—C43	1.371 (8)
C8—H8	0.9500	C42—H42	0.9500
C9—C10	1.398 (7)	C43—C44	1.402 (7)
C10—C11	1.408 (8)	C43—H43	0.9500
C10—C27	1.487 (7)	C44—H44	0.9500
C11—C12	1.434 (7)	C45—C50	1.396 (15)
C12—C13	1.347 (7)	C45—C46	1.434 (13)
C12—H12	0.9500	C46—C47	1.357 (13)
C13—C14	1.446 (7)	C47—C48	1.361 (13)
C13—H13	0.9500	C48—C49	1.390 (13)
C14—C15	1.395 (7)	C48—H48	0.9500
C15—C16	1.391 (7)	C49—C50	1.374 (13)
C15—C33	1.502 (7)	C45'—C46'	1.34 (7)
C16—C17	1.441 (7)	C45'—C50'	1.39 (5)
C17—C18	1.343 (7)	C46'—C47'	1.38 (6)
C17—H17	0.9500	C47'—C48'	1.35 (5)
C18—C19	1.438 (8)	C48'—C49'	1.40 (6)
C18—H18	0.9500	C48'—H48'	0.9500
C19—C20	1.395 (7)	C49'—C50'	1.48 (6)
			
O1—Fe1—N2	100.8 (2)	C23—C22—C21	121.2 (5)
O1—Fe1—N1	105.6 (2)	C23—C22—H22	119.4
N2—Fe1—N1	87.14 (17)	C21—C22—H22	119.4
O1—Fe1—N4	106.8 (2)	C22—C23—C24	119.3 (5)
N2—Fe1—N4	152.3 (2)	C22—C23—H23	120.3
N1—Fe1—N4	86.76 (16)	C24—C23—H23	120.3
O1—Fe1—N3	101.1 (2)	C25—C24—C23	120.0 (6)
N2—Fe1—N3	86.62 (17)	C25—C24—H24	120.0
N1—Fe1—N3	153.3 (2)	C23—C24—H24	120.0
N4—Fe1—N3	86.80 (17)	C24—C25—C26	120.9 (5)
C45—O1—Fe1	121.4 (7)	C24—C25—H25	119.6
O1'—Fe1'—N4	106.6 (7)	C26—C25—H25	119.6
O1'—Fe1'—N3	112.6 (7)	C25—C26—C21	120.1 (5)
N4—Fe1'—N3	87.0 (2)	C25—C26—H26	120.0
O1'—Fe1'—N2	109.8 (7)	C21—C26—H26	120.0
N4—Fe1'—N2	143.2 (3)	C32—C27—C28	118.2 (5)
N3—Fe1'—N2	84.2 (2)	C32—C27—C10	120.7 (5)
O1'—Fe1'—N1	104.7 (7)	C28—C27—C10	121.1 (5)
N4—Fe1'—N1	83.9 (2)	C29—C28—C27	119.9 (5)
N3—Fe1'—N1	142.7 (3)	C29—C28—H28	120.0
N2—Fe1'—N1	81.8 (2)	C27—C28—H28	120.0
C45'—O1'—Fe1'	123 (3)	C30—C29—C28	120.8 (5)
C4—N1—C1	105.6 (4)	C30—C29—H29	119.6
C4—N1—Fe1	126.6 (3)	C28—C29—H29	119.6
C1—N1—Fe1	126.6 (3)	C29—C30—C31	119.5 (5)
C4—N1—Fe1'	125.2 (4)	C29—C30—H30	120.3
C1—N1—Fe1'	122.8 (3)	C31—C30—H30	120.3
C9—N2—C6	105.5 (4)	C32—C31—C30	120.4 (5)
C9—N2—Fe1	126.6 (3)	C32—C31—H31	119.8
C6—N2—Fe1	126.9 (3)	C30—C31—H31	119.8
C9—N2—Fe1'	122.4 (4)	C31—C32—C27	121.1 (5)
C6—N2—Fe1'	125.3 (3)	C31—C32—H32	119.4
C11—N3—C14	106.0 (4)	C27—C32—H32	119.4
C11—N3—Fe1'	126.5 (4)	C34—C33—C38	118.8 (5)
C14—N3—Fe1'	122.9 (3)	C34—C33—C15	120.6 (5)
C11—N3—Fe1	126.2 (4)	C38—C33—C15	120.6 (5)
C14—N3—Fe1	125.5 (3)	C35—C34—C33	121.2 (5)
C19—N4—C16	106.1 (4)	C35—C34—H34	119.4
C19—N4—Fe1'	126.7 (4)	C33—C34—H34	119.4
C16—N4—Fe1'	122.9 (3)	C34—C35—C36	120.1 (5)
C19—N4—Fe1	125.7 (4)	C34—C35—H35	120.0
C16—N4—Fe1	125.5 (3)	C36—C35—H35	120.0
C20—C1—N1	125.4 (5)	C35—C36—C37	119.0 (5)
C20—C1—C2	125.2 (5)	C35—C36—H36	120.5
N1—C1—C2	109.4 (5)	C37—C36—H36	120.5
C3—C2—C1	107.6 (5)	C38—C37—C36	121.3 (5)
C3—C2—H2	126.2	C38—C37—H37	119.4
C1—C2—H2	126.2	C36—C37—H37	119.4
C2—C3—C4	107.0 (5)	C37—C38—C33	119.5 (5)
C2—C3—H3	126.5	C37—C38—H38	120.2
C4—C3—H3	126.5	C33—C38—H38	120.2
N1—C4—C5	125.6 (5)	C40—C39—C44	118.7 (5)
N1—C4—C3	110.3 (5)	C40—C39—C20	121.8 (5)
C5—C4—C3	124.0 (5)	C44—C39—C20	119.6 (5)
C4—C5—C6	124.4 (5)	C39—C40—C41	121.4 (5)
C4—C5—C21	118.5 (5)	C39—C40—H40	119.3
C6—C5—C21	117.1 (5)	C41—C40—H40	119.3
N2—C6—C5	125.3 (5)	C40—C41—C42	119.6 (5)
N2—C6—C7	110.0 (4)	C40—C41—H41	120.2
C5—C6—C7	124.7 (5)	C42—C41—H41	120.2
C8—C7—C6	107.3 (5)	C43—C42—C41	120.1 (5)
C8—C7—H7	126.3	C43—C42—H42	119.9
C6—C7—H7	126.3	C41—C42—H42	119.9
C7—C8—C9	107.1 (5)	C42—C43—C44	119.8 (5)
C7—C8—H8	126.5	C42—C43—H43	120.1
C9—C8—H8	126.5	C44—C43—H43	120.1
N2—C9—C10	126.4 (5)	C39—C44—C43	120.4 (5)
N2—C9—C8	110.1 (4)	C39—C44—H44	119.8
C10—C9—C8	123.5 (5)	C43—C44—H44	119.8
C9—C10—C11	123.6 (5)	O1—C45—C50	122.9 (9)
C9—C10—C27	118.7 (5)	O1—C45—C46	124.0 (8)
C11—C10—C27	117.7 (5)	C50—C45—C46	113.0 (9)
N3—C11—C10	125.6 (5)	C47—C46—F1	121.5 (9)
N3—C11—C12	110.6 (5)	C47—C46—C45	122.2 (10)
C10—C11—C12	123.8 (5)	F1—C46—C45	116.3 (8)
C13—C12—C11	106.9 (5)	C46—C47—C48	123.3 (10)
C13—C12—H12	126.5	C46—C47—F2	117.3 (10)
C11—C12—H12	126.5	C48—C47—F2	119.4 (9)
C12—C13—C14	107.8 (5)	C47—C48—C49	116.6 (8)
C12—C13—H13	126.1	C47—C48—H48	121.7
C14—C13—H13	126.1	C49—C48—H48	121.7
N3—C14—C15	126.1 (5)	F3—C49—C50	120.1 (9)
N3—C14—C13	108.8 (5)	F3—C49—C48	118.9 (8)
C15—C14—C13	125.2 (5)	C50—C49—C48	121.0 (9)
C16—C15—C14	124.1 (5)	F4—C50—C49	117.9 (9)
C16—C15—C33	117.2 (5)	F4—C50—C45	118.1 (7)
C14—C15—C33	118.7 (5)	C49—C50—C45	123.9 (10)
C15—C16—N4	126.4 (5)	O1'—C45'—C46'	121 (4)
C15—C16—C17	124.7 (5)	O1'—C45'—C50'	122 (4)
N4—C16—C17	108.9 (5)	C46'—C45'—C50'	117 (5)
C18—C17—C16	108.1 (5)	C45'—C46'—C47'	126 (5)
C18—C17—H17	126.0	C45'—C46'—F1'	123 (4)
C16—C17—H17	126.0	C47'—C46'—F1'	112 (4)
C17—C18—C19	107.5 (5)	C48'—C47'—C46'	122 (4)
C17—C18—H18	126.2	C48'—C47'—F2'	117 (4)
C19—C18—H18	126.2	C46'—C47'—F2'	122 (4)
N4—C19—C20	126.0 (5)	C47'—C48'—C49'	116 (4)
N4—C19—C18	109.4 (5)	C47'—C48'—H48'	122.0
C20—C19—C18	124.6 (5)	C49'—C48'—H48'	122.0
C1—C20—C19	124.3 (5)	F3'—C49'—C48'	120 (5)
C1—C20—C39	117.1 (5)	F3'—C49'—C50'	118 (4)
C19—C20—C39	118.5 (5)	C48'—C49'—C50'	122 (4)
C22—C21—C26	118.6 (5)	F4'—C50'—C45'	125 (4)
C22—C21—C5	120.2 (5)	F4'—C50'—C49'	117 (4)
C26—C21—C5	121.2 (5)	C45'—C50'—C49'	118 (4)
			
N4—Fe1'—O1'—C45'	−25 (3)	N1—C1—C20—C39	−178.2 (5)
N3—Fe1'—O1'—C45'	−119 (3)	C2—C1—C20—C39	3.0 (8)
N2—Fe1'—O1'—C45'	149 (3)	N4—C19—C20—C1	1.6 (9)
N1—Fe1'—O1'—C45'	63 (3)	C18—C19—C20—C1	−179.4 (5)
C4—N1—C1—C20	−178.8 (5)	N4—C19—C20—C39	−178.3 (5)
Fe1—N1—C1—C20	12.8 (7)	C18—C19—C20—C39	0.7 (8)
Fe1'—N1—C1—C20	−25.6 (7)	C4—C5—C21—C22	64.0 (7)
C4—N1—C1—C2	0.2 (6)	C6—C5—C21—C22	−114.9 (5)
Fe1—N1—C1—C2	−168.2 (4)	C4—C5—C21—C26	−118.4 (6)
Fe1'—N1—C1—C2	153.4 (4)	C6—C5—C21—C26	62.7 (7)
C20—C1—C2—C3	179.7 (5)	C26—C21—C22—C23	−0.5 (7)
N1—C1—C2—C3	0.7 (6)	C5—C21—C22—C23	177.1 (5)
C1—C2—C3—C4	−1.3 (6)	C21—C22—C23—C24	−0.4 (8)
C1—N1—C4—C5	177.7 (5)	C22—C23—C24—C25	0.8 (8)
Fe1—N1—C4—C5	−13.9 (8)	C23—C24—C25—C26	−0.3 (8)
Fe1'—N1—C4—C5	25.4 (8)	C24—C25—C26—C21	−0.7 (8)
C1—N1—C4—C3	−1.0 (6)	C22—C21—C26—C25	1.1 (8)
Fe1—N1—C4—C3	167.4 (4)	C5—C21—C26—C25	−176.6 (5)
Fe1'—N1—C4—C3	−153.4 (4)	C9—C10—C27—C32	113.7 (6)
C2—C3—C4—N1	1.5 (6)	C11—C10—C27—C32	−64.5 (7)
C2—C3—C4—C5	−177.3 (5)	C9—C10—C27—C28	−66.0 (7)
N1—C4—C5—C6	−2.8 (9)	C11—C10—C27—C28	115.7 (6)
C3—C4—C5—C6	175.8 (5)	C32—C27—C28—C29	1.3 (8)
N1—C4—C5—C21	178.4 (5)	C10—C27—C28—C29	−179.0 (5)
C3—C4—C5—C21	−3.0 (8)	C27—C28—C29—C30	0.9 (9)
C9—N2—C6—C5	−179.5 (5)	C28—C29—C30—C31	−2.8 (9)
Fe1—N2—C6—C5	11.4 (7)	C29—C30—C31—C32	2.5 (9)
Fe1'—N2—C6—C5	−28.3 (7)	C30—C31—C32—C27	−0.3 (9)
C9—N2—C6—C7	0.0 (6)	C28—C27—C32—C31	−1.6 (8)
Fe1—N2—C6—C7	−169.1 (3)	C10—C27—C32—C31	178.6 (5)
Fe1'—N2—C6—C7	151.2 (4)	C16—C15—C33—C34	116.0 (5)
C4—C5—C6—N2	4.1 (8)	C14—C15—C33—C34	−62.0 (7)
C21—C5—C6—N2	−177.1 (5)	C16—C15—C33—C38	−62.9 (7)
C4—C5—C6—C7	−175.3 (5)	C14—C15—C33—C38	119.1 (6)
C21—C5—C6—C7	3.5 (8)	C38—C33—C34—C35	1.8 (8)
N2—C6—C7—C8	−0.1 (6)	C15—C33—C34—C35	−177.1 (5)
C5—C6—C7—C8	179.4 (5)	C33—C34—C35—C36	0.4 (8)
C6—C7—C8—C9	0.1 (6)	C34—C35—C36—C37	−1.7 (8)
C6—N2—C9—C10	179.9 (5)	C35—C36—C37—C38	0.9 (8)
Fe1—N2—C9—C10	−11.0 (8)	C36—C37—C38—C33	1.3 (8)
Fe1'—N2—C9—C10	27.6 (7)	C34—C33—C38—C37	−2.6 (8)
C6—N2—C9—C8	0.1 (6)	C15—C33—C38—C37	176.3 (5)
Fe1—N2—C9—C8	169.2 (3)	C1—C20—C39—C40	−116.5 (6)
Fe1'—N2—C9—C8	−152.2 (4)	C19—C20—C39—C40	63.5 (7)
C7—C8—C9—N2	−0.2 (6)	C1—C20—C39—C44	63.6 (7)
C7—C8—C9—C10	−180.0 (5)	C19—C20—C39—C44	−116.4 (6)
N2—C9—C10—C11	−4.5 (9)	C44—C39—C40—C41	−1.6 (8)
C8—C9—C10—C11	175.3 (5)	C20—C39—C40—C41	178.5 (5)
N2—C9—C10—C27	177.4 (5)	C39—C40—C41—C42	−0.4 (8)
C8—C9—C10—C27	−2.8 (8)	C40—C41—C42—C43	1.7 (8)
C14—N3—C11—C10	−178.4 (5)	C41—C42—C43—C44	−0.9 (9)
Fe1'—N3—C11—C10	−22.3 (8)	C40—C39—C44—C43	2.3 (8)
Fe1—N3—C11—C10	18.2 (8)	C20—C39—C44—C43	−177.8 (5)
C14—N3—C11—C12	1.6 (6)	C42—C43—C44—C39	−1.1 (9)
Fe1'—N3—C11—C12	157.7 (4)	Fe1—O1—C45—C50	−84.1 (13)
Fe1—N3—C11—C12	−161.7 (4)	Fe1—O1—C45—C46	93.5 (12)
C9—C10—C11—N3	0.6 (9)	O1—C45—C46—C47	−175.9 (10)
C27—C10—C11—N3	178.7 (5)	C50—C45—C46—C47	1.9 (15)
C9—C10—C11—C12	−179.5 (5)	O1—C45—C46—F1	2.6 (16)
C27—C10—C11—C12	−1.4 (8)	C50—C45—C46—F1	−179.6 (9)
N3—C11—C12—C13	−2.0 (6)	F1—C46—C47—C48	−179.6 (8)
C10—C11—C12—C13	178.0 (5)	C45—C46—C47—C48	−1.2 (15)
C11—C12—C13—C14	1.5 (6)	F1—C46—C47—F2	1.6 (13)
C11—N3—C14—C15	178.6 (5)	C45—C46—C47—F2	−179.9 (9)
Fe1'—N3—C14—C15	21.4 (8)	C46—C47—C48—C49	0.7 (14)
Fe1—N3—C14—C15	−17.9 (8)	F2—C47—C48—C49	179.5 (8)
C11—N3—C14—C13	−0.6 (6)	C47—C48—C49—F3	178.0 (8)
Fe1'—N3—C14—C13	−157.8 (4)	C47—C48—C49—C50	−1.2 (12)
Fe1—N3—C14—C13	162.9 (4)	F3—C49—C50—F4	1.3 (14)
C12—C13—C14—N3	−0.6 (6)	C48—C49—C50—F4	−179.5 (9)
C12—C13—C14—C15	−179.9 (5)	F3—C49—C50—C45	−177.0 (11)
N3—C14—C15—C16	2.5 (9)	C48—C49—C50—C45	2.2 (16)
C13—C14—C15—C16	−178.4 (5)	O1—C45—C50—F4	−2.9 (18)
N3—C14—C15—C33	−179.6 (5)	C46—C45—C50—F4	179.3 (10)
C13—C14—C15—C33	−0.5 (8)	O1—C45—C50—C49	175.4 (11)
C14—C15—C16—N4	−2.5 (8)	C46—C45—C50—C49	−2.4 (17)
C33—C15—C16—N4	179.5 (5)	Fe1'—O1'—C45'—C46'	83 (6)
C14—C15—C16—C17	177.0 (5)	Fe1'—O1'—C45'—C50'	−90 (6)
C33—C15—C16—C17	−1.0 (7)	O1'—C45'—C46'—C47'	−173 (6)
C19—N4—C16—C15	−179.5 (5)	C50'—C45'—C46'—C47'	0 (9)
Fe1'—N4—C16—C15	−21.5 (7)	O1'—C45'—C46'—F1'	4 (9)
Fe1—N4—C16—C15	18.0 (7)	C50'—C45'—C46'—F1'	177 (5)
C19—N4—C16—C17	1.0 (6)	C45'—C46'—C47'—C48'	−3 (9)
Fe1'—N4—C16—C17	159.0 (4)	F1'—C46'—C47'—C48'	−180 (4)
Fe1—N4—C16—C17	−161.5 (3)	C45'—C46'—C47'—F2'	178 (5)
C15—C16—C17—C18	179.7 (5)	F1'—C46'—C47'—F2'	1 (7)
N4—C16—C17—C18	−0.7 (6)	C46'—C47'—C48'—C49'	2 (6)
C16—C17—C18—C19	0.2 (6)	F2'—C47'—C48'—C49'	−179 (3)
C16—N4—C19—C20	178.3 (5)	C47'—C48'—C49'—F3'	−179 (4)
Fe1'—N4—C19—C20	21.4 (8)	C47'—C48'—C49'—C50'	1 (6)
Fe1—N4—C19—C20	−19.3 (8)	O1'—C45'—C50'—F4'	−13 (8)
C16—N4—C19—C18	−0.9 (6)	C46'—C45'—C50'—F4'	174 (5)
Fe1'—N4—C19—C18	−157.8 (4)	O1'—C45'—C50'—C49'	175 (5)
Fe1—N4—C19—C18	161.6 (3)	C46'—C45'—C50'—C49'	2 (8)
C17—C18—C19—N4	0.4 (6)	F3'—C49'—C50'—F4'	4 (6)
C17—C18—C19—C20	−178.7 (5)	C48'—C49'—C50'—F4'	−175 (4)
N1—C1—C20—C19	1.9 (9)	F3'—C49'—C50'—C45'	177 (4)
C2—C1—C20—C19	−177.0 (5)	C48'—C49'—C50'—C45'	−3 (7)
